# Genetically Targeted Connectivity Tracing Excludes Dopaminergic Inputs to the Interpeduncular Nucleus from the Ventral Tegmentum and Substantia Nigra

**DOI:** 10.1523/ENEURO.0127-21.2021

**Published:** 2021-06-18

**Authors:** Nailyam Nasirova, Lely A. Quina, Shoshana Novik, Eric E. Turner

**Affiliations:** 1 Center for Integrative Brain Research, Seattle Children’s Research Institute, 1900 Ninth Avenue, Seattle, WA 98101; 2 Department of Psychiatry and Behavioral Sciences, University of Washington, Seattle, WA 98195

**Keywords:** dopamine, habenula, interpeduncular nucleus, nicotine, substantia nigra, ventral tegmental area

## Abstract

The “habenulopeduncular system” consists of the medial habenula (MHb) and its principal target of innervation, the interpeduncular nucleus (IP). Neurons in the ventral MHb (MHbV) express acetylcholine along with glutamate, and both the MHb and IP are rich in nicotinic acetylcholine receptors. Much of the work on this system has focused on nicotinic mechanisms and their clinical implications for nicotine use, particularly because the IP expresses the α5 nicotinic receptor subunit, encoded by the CHRNA5 gene, which is genetically linked to smoking risk. A working model has emerged in which nicotine use may be determined by the balance of reinforcement mediated in part by nicotine effects on dopamine reward pathways, and an aversive “brake” on nicotine consumption encoded in the MHb-IP pathway. However, recent work has proposed that the IP also receives direct dopaminergic input from the ventral tegmental area (VTA). If correct, this would significantly impact the prevailing model of IP function. Here, we have used *Chrna5^Cre^* mice to perform rabies virus-mediated retrograde tracing of global inputs to the IP. We have also used Cre-dependent adeno-associated virus (AAV) anterograde tracing using *Slc6a3^Cre^* (*DAT^Cre^*) mice to map VTA dopaminergic efferents, and we have examined tract-tracing data using other transgenic models for dopaminergic neurons available in a public database. Consistent with the existing literature using non-genetic tracing methods, none of these experiments show a significant anatomic connection from the VTA or substantia nigra (SN) to the IP, and thus do not support a model of direct dopaminergic input to the habenulopeduncular system.

## Significance Statement

The interpeduncular nucleus (IP) is the central node in a descending brain pathway linking the medial habenula (MHb) of the epithalamus to the mesopontine tegmentum. Neurons in this “habenulopeduncular pathway” express unusual nicotinic acetylcholine receptors and are thought to play a role in behavioral responses to nicotine. In particular, the IP expresses the α5 nicotinic receptor, human variants of which confer increased risk of smoking. Recently it has been reported that inputs from midbrain dopaminergic neurons to the IP also influence behavior. Here, we have used a mouse genetic model to map specific inputs to the α5-expressing neurons in the IP. Contrary to the published reports, we do not identify dopaminergic inputs to the IP.

## Introduction

The habenula is a bilateral epithalamic structure that provides a major link between the limbic forebrain and nuclei in the mesopontine tegmentum, including the ventral tegmental area (VTA), the rostromedial tegmental nucleus (RMTg), the interpeduncular nucleus (IP), and the raphe nuclei. The two major divisions of the habenula, the medial habenula (MHb) and lateral habenula (LHb), appear to serve largely distinct, parallel pathways (Fakhoury, 2018; Metzger et al., 2021). Although the MHb and LHb efferents initially travel via a single prominent tract, the fasciculus retroflexus, their outputs terminate in distinct tegmental areas. The MHb predominantly innervates the IP, with glutamatergic/cholinergic fibers from the ventral MHb (MHbV), and glutamatergic/peptidergic fibers from the dorsal MHb (MHbD), connecting to specific IP subnuclei to form the “habenulopeduncular system” (Quina et al., 2017). Although the precise function of the IP is not well understood, much of the interest in MHb-IP pathway has been driven by the expression of specific nicotinic acetylcholine receptors in the MHbV and IP (Quina et al., 2009; Hsu et al., 2013), the dual glutamatergic/cholinergic nature of MHbV neurons that project to the IP (Qin and Luo, 2009; Hsu et al., 2013; Frahm et al., 2015), the effect of nicotine on signaling in and between the MHbV and IP (Ables et al., 2017; Morton et al., 2018; Wolfman et al., 2018; Arvin et al., 2019), and its possible relevance to the balance of nicotine reward and aversion that regulates smoking (Fowler and Kenny, 2014; McLaughlin et al., 2017; Lee et al., 2019).

The IP expresses particularly high levels of the α5 nicotinic receptor subunit, product of the *Chrna5* gene (Boulter et al., 1990; Hsu et al., 2013). The strongest known genetic risk for increased tobacco consumption is associated with certain haplotypes that occur at high frequency in European populations, and contain a nonsynonymous CHRNA5 polymorphism, CHRNA5(D398N) (Berrettini and Doyle, 2012; Lassi et al., 2016), which appears to reduce the function of nicotinic receptors into which this variant α5 subunit is incorporated (Kuryatov et al., 2011; Sciaccaluga et al., 2015). Although in humans and rodents the *Chrna5* gene locus is part of a conserved cluster with two other nicotinic receptor genes, *Chrna3* and *Chrnb4*, transgenic mice have been devised for expression of Cre recombinase regulated by the *Chrna5* locus without over-expression of the other receptors in this cluster, and have been used to show that optogenetic activation of α5-expressing neurons in the IP is aversive if mice have been previously exposed to nicotine (Morton et al., 2018).

In contrast, the best characterized outputs of the LHb are projections to the VTA, both directly and via GABAergic intermediaries in the RMTg (Petzel et al., 2017; Li et al., 2019), and projections to the mesopontine raphe (Sego et al., 2014; Tchenio et al., 2016; Quina et al., 2017, 2020). Reciprocal connections between the LHb and both dopaminergic and non-dopaminergic neurons in the VTA have been demonstrated (Root et al., 2014; Zahm and Root, 2017). Thus, in terms of neurotransmitter systems, work on the LHb has emphasized regulation of dopamine and serotonin (Metzger et al., 2021).

Given these largely parallel pathways, little attention has been paid to the possibility of a direct interaction between midbrain DA neurons and the MHb-IP pathway. Classic tract-tracing studies using histochemical techniques have shown that the IP integrates inputs from many brain regions, including tegmental afferents from the median raphe, nucleus incertus (NI), and laterodorsal tegmentum, but have not identified inputs from the VTA or substantia nigra (SN; Marchand et al., 1980; Hamill and Lenn, 1984; Groenewegen et al., 1986; Shibata et al., 1986; Lima et al., 2017; Bueno et al., 2019). Despite these studies, it has been recently proposed that the IP receives functional dopaminergic inputs from the VTA, which modulate nicotine withdrawal-induced anxiety and novelty signaling in behavioral tests of social familiarity (Zhao-Shea et al., 2015; Molas et al., 2017). Such a direct interaction between DA neurons and the MHb-IP pathway, if confirmed, could shift the paradigm for how this pathway functions in reinforcement and other behaviors. Here, however, we have used rabies-virus mediated retrograde tracing in *Chrna5^Cre^* mice, and virally-mediated anterograde tracing in multiple transgenic models, and find no evidence for an anatomic connection between midbrain DA neurons and the IP.

## Materials and Methods

### Mouse strains

Transgenic targeting of the IP was achieved with a Chrna5-BAC-Cre transgenic line (*Chrna5^Cre^*; Morton et al., 2018). Transgenic reporting of Cre-expression was achieved by crossing *Chrna5^Cre^* mice with the Cre-dependent ZsGreen reporter strain *Gt(ROSA).26Sor^tm6(CAG-ZsGreen1)Hze/J^* (*Ai6*, Jax #007906; Madisen et al., 2010). Viral reporters were targeted to tegmental DA neurons using the transgenic line Slc6a3^tm1.1(cre)Bkmn/J^ (*DAT^Cre^*, Jax #006660; Bäckman et al., 2006). All strains were maintained on a C57BL/6NCrl genetic background (Charles River). Adult mice of both sexes were used in the experiments. Data for three other transgenic lines were obtained from the Allen Mouse Brain Connectivity Atlas, as described below.

### Immunofluorescence and *in situ* hybridization

Mouse brain tissue was prepared by fixation via transcardial perfusion with 4% paraformaldehyde. Brains were then removed and equilibrated in graded sucrose solutions, frozen at −8°C in OCT solution, and cryosectioned at 25 μm for fluorescence/immunofluorescence imaging. Tissue processed in this way was suitable for imaging of endogenous protein fluorescence, immunofluorescence, and fluorescence *in situ* hybridization (FISH). Tyrosine hydroxylase (TH) immunoreactivity was detected using rabbit anti-TH (AB152, EMD Millipore, RRID:AB_390204). Multi-channel FISH was performed with the RNAscope Multiplex Fluorescent V2 kit, according to the manufacturer’s instructions (Advanced Cell Diagnostics). The probes used included: EGFP, #400281-C2 (channel 2) and Mm-Rln3-C1 (channel 1).

### Anterograde tracing

The targeted coordinates for each anterograde or retrograde tracing injection were based on a standard atlas (Paxinos and Franklin, 2001). For anterograde tracing of VTA efferents, 100 nl of viral stock was pressure injected at coordinates: AP −3.4, ML 0.5, DV 4.5. Anterograde tract tracing data derived from the Allen Mouse Brain Connectivity Atlas were generated using iontophoretic injection of AAV, and detailed methods have been published in conjunction with the Atlas (Harris et al., 2012; Oh et al., 2014). Animals were fixed by transcardial perfusion with 4% paraformaldehyde at 14–21 d after injection and processed as described above. Anterograde tracing was performed using Cre-activated (FLEX) adeno-associated virus (AAV; capsid strain 1). Enhanced labeling of presynaptic areas was performed by expression of a synaptophysin-EGFP fusion protein (sypGFP). The plasmid pCAG.Flex.sypEGFP.WPRE (“FLEX-sypGFP”) was constructed by replacing the EGFP moiety of pCAG-FLEX-EGFP-WPRE (Addgene #51 502) with the sypEGFP construct from phSyn1(S)-FLEX-tdTomato-T2A-SypEGFP-WPRE (Addgene #51509) by Julie Harris, Karla Hirokawa and Hong Gu of the Allen Institute for Brain Science (gift of Julie Harris).

### Anterograde tract tracing: database information

The Allen Mouse Brain Connectivity Atlas provides a searchable database of brain-wide AAV tract-tracing datasets using wild-type and Cre-recombinase expressing mouse strains (https://connectivity.brain-map.org/). A source structure database search was performed for the VTA, and a target structure search was performed for the IP. Three informative cases (experiments) were identified with the VTA as the fiber source, using three different Cre-drivers related to monoaminergic transmission to specifically target the VTA: (1) *TH-IRES-CreER* (JAX #008532, *TH^Cre^*; Rotolo et al., 2008), experiment 156314762, published online 10/04/2012; (2) *Slc6a3-Cre* (*DAT^Cre^*; Zhuang et al., 2005), experiment 160539283, published 03/07/2013; (3) *Slc18a2-Cre_OZ14* (GENSAT BAC-Cre, *VMAT2^Cre^*; Gong et al., 2007), experiment 292958638, published 03/06/2014.

The online publication dates of the Allen Connectivity Atlas datasets were provided by Hongkui Zeng, Allen Institute for Brain Science, Seattle, WA.

### Retrograde tracing

For retrograde tracing from a genetically defined cell population, the helper virus AAV1-Syn-DIO-TVA66T-dTom-CVS N2cG, (AAV1-N2cG) a tricistronic virus which expresses the pseudotyping receptor TVA, tdTomato, and the rabies glycoprotein G (Lo et al., 2019) was injected by pressure injection into the IP of *Chrna5^Cre^* mice, followed by the rabies virus EnvA CVS-N2cΔG-histone-eGFP (RV-GFP), injected 21 d later into the same location. The injection coordinates were: AP 3.4, ML 0.0, DV 5.0, and the injected volume was 200 nl for the helper virus and 300 nl for the rabies virus. AAV1 and RV for retrograde tracing were the gifts of Shenqin Yao and Ali Cetin (Allen Institute for Brain Science). Further details regarding rabies reagents are available on request from Shenqin Yao and Ali Cetin. Mice were euthanized 10 d later and the brains were processed as described above to visualize the nuclear GFP signal, or virally expressed GFP mRNA using FISH, in presynaptic neurons.

## Results

**Figure 1.**
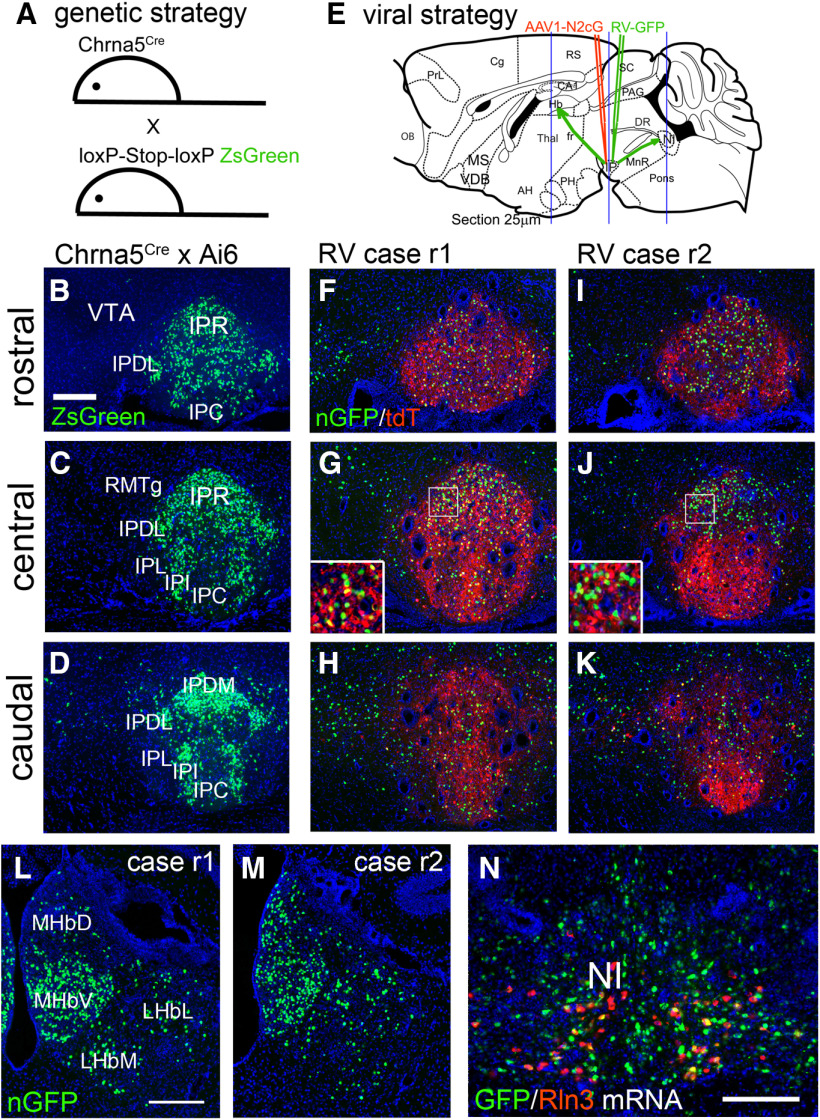
Genetic and viral strategy for transsynaptic labeling of IP afferents. ***A***, A genetic strategy for labeling Cre-expressing neurons in the IP of *Chrna5^Cre^* mice. *Chrna5^Cre^* mice were interbred with the mouse strain Ai6, which conditionally expresses the fluorophore ZsGreen (Materials and Methods). ***B–D***, ZsGreen expression in the rostral, central, and caudal IP of *Chrna5^Cre^*, *Ai6* compound heterozygous mice, generated as shown in ***A***. ***E***, RV transsynaptic labeling strategy. A helper virus AAV1-N2cG was injected into the IP of *Chrna5^Cre^* mice, followed by RV three weeks later (Materials and Methods). The habenula and IP were examined for the expression of nuclear GFP (nGFP) expressed by RV. Blue lines indicate the planes of section for the habenula, IP, and NI (rostral to caudal). ***F–K***, Imaging of nGFP expressed by RV and cytoplasmic tdTomato (tdT) expressed by the AAV helper virus in the rostral (***F***, ***I***), central (***G***, ***J***), and caudal (***H***, ***K***) IP of two injected cases, r1 and r2 (retrograde 1 and 2). Insets in ***G***, ***J*** show higher magnification of the boxed area. ***L*, *M***, Expression of RV nGFP transsynaptic label in the habenula. Coronal sections correspond to bregma −1.7 in a standard atlas (Paxinos and Franklin, 2001). ***N***, Dual-label FISH for RV-expressed GFP mRNA and Rln3 mRNA in the NI of injected Case r1. IP, interpeduncular nucleus; IPC, central part; IPDL, dorsolateral part; IPDM, dorsomedial part; IPI, intermediate part; IPL, lateral part; IPR, rostral part; LHbL, lateral habenula, lateral part; LHbM, lateral habenula, medial part; MHbD, medial habenula, dorsal part; MHbV, medial habenula, ventral part; NI, nucleus incertus; RMTg, rostromedial tegmental nucleus; VTA, ventral tegmental area. Scale bar: 200 μm (***B***, ***L***, ***N***).

RV-mediated retrograde tracing is usually targeted by the specific expression of Cre-recombinase in the postsynaptic neurons of interest. In order to trace specific inputs to the IP, we used a well-characterized mouse BAC transgenic strain, *Chrna5^Cre^*, which expresses Cre recombinase in most neurons of the IP, and in a small population of GABAergic neurons in the adjacent median raphe (Morton et al., 2018). Although α5 mRNA is expressed in some neurons of the VTA, the regulatory sequences in this BAC transgene do not target VTA neurons, leading to high local specificity of Cre expression in the IP. This specificity of Cre expression was verified by crossing *Chrna5^Cre^* mice with a genetic reporter strain, Ai6 (Fig. 1*A*; Materials and Methods), which allows Cre-dependent expression of the reporter ZsGreen in targeted cells. The ZsGreen reporter was expressed abundantly in all of the IP subnuclei, except the lateral subnucleus, which showed sparse labeling (Fig. 1*B–D*), whereas labeling was not observed in the VTA (Fig. 1*B*).

A cohort of five *Chrna5^Cre^* mice were used for retrograde tracing. These mice were first injected in the IP with the “helper” virus AAV1-N2cG, which is a tricistronic vector expressing pseudotyping receptor TVA, tdTomato, and the rabies glycoprotein G (Materials and Methods). Three weeks later the same site was injected with RV expressing nuclear GFP (nGFP), and after 10 d the mice were killed, and brains were processed for localization of RV-nGFP and other markers. The two cases with the most comprehensive labeling of the targeted IP neurons were chosen for detailed characterization (Cases r1, r2; Fig. 1*F–K*). As expected, tdTomato from AAV1-N2cG and nGFP-labeled “starter cells” capable of infecting IP afferents were observed in all IP subnuclei, but were sparse in IPL. In order to determine the efficiency of RV-mediated retrograde labeling, we examined RV-nGFP expression in two areas known to have strong inputs to the IP, the habenula (Fig. 1*L*,*M*, ref) and the NI (Fig. 1*N*; Nasirova et al., 2020). RV-Case r1 resulted in strong labeling of the MHbV, and partial labeling of the MHbD and LHb. RV-Case r2 resulted in strong labeling of both MHbV and MHbD, as well as partial labeling of LHb. More intense labeling of MHbD in Cases r2 is expected, because the injected area is more caudally positioned in the IP, where MHbD fibers are known to cross the midline and terminate (Quina et al., 2017). The NI of Case r1 was examined for expression of RV-GFP mRNA together with mRNA for the characteristic NI neuropeptide, relaxin-3 (Rln3). Consistent with prior studies using anterograde tracing of Rln3-positive and Rln3-negative NI neurons (Nasirova et al., 2020), most of the RV-nGFP labeled neurons in the NI did not express Rln3. Together these two RV-injected cases appear to give nearly complete coverage of the α5-expressing cell population in the IP.

**Figure 2.**
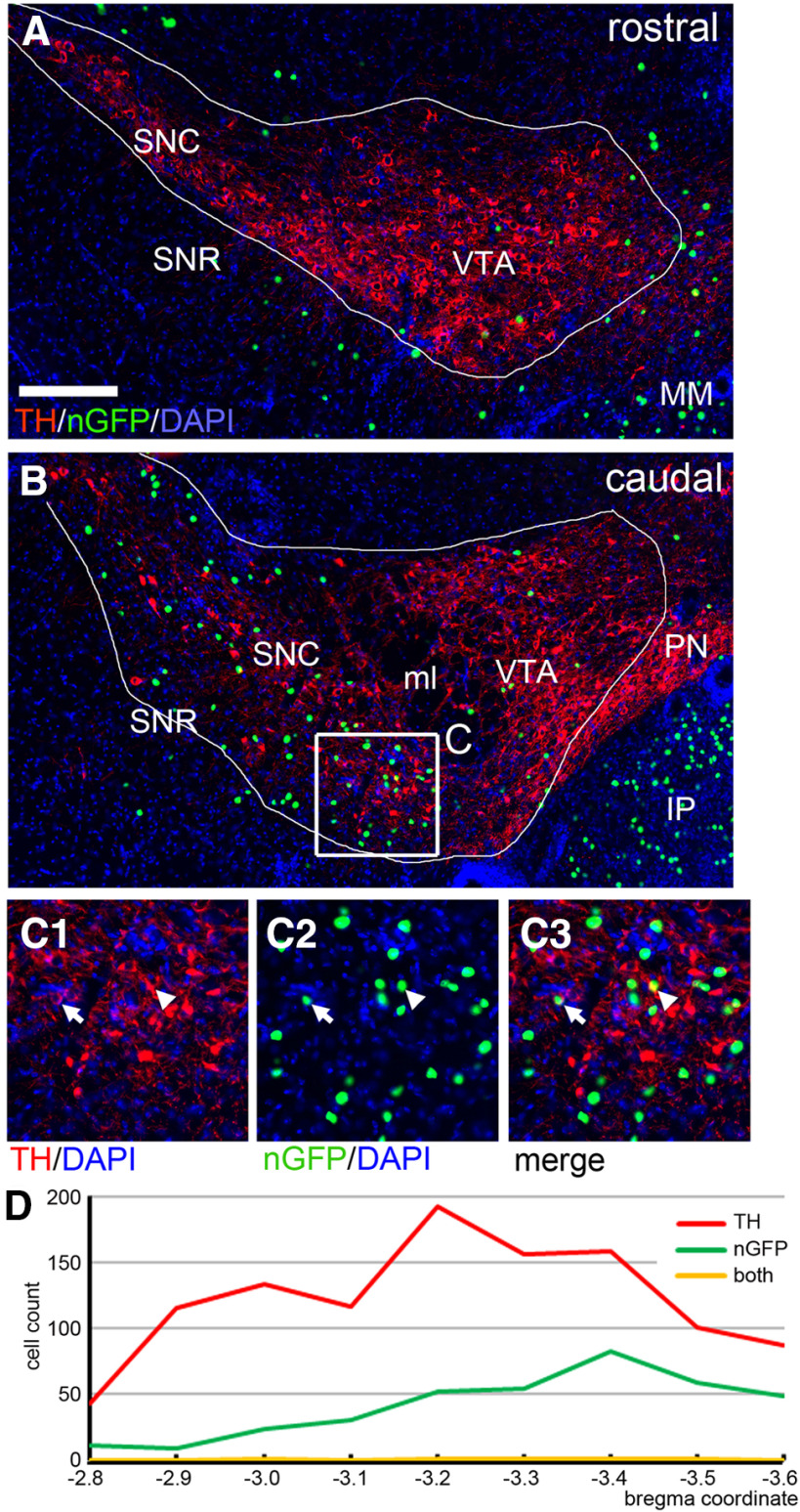
RV-mediated retrograde tracing of projections from the VTA and SN to the IP. Inputs to the IP were retrogradely labeled with RV as described in Figure 1 (Case r1), producing a nuclear GFP label in presynaptic neurons. Serial 25-μm sections were examined at 100-μm intervals through the VTA and SN, at levels from bregma −2.8 to bregma −3.6 in a standard atlas (Paxinos and Franklin, 2001), and imaged as Z-stacks. DA neurons were labeled by immunostaining for TH, and all cells were counterstained for nucleic acids with DAPI. TH-positive neurons were counted if the TH immunostaining formed a cytoplasmic circle or semicircle around a nuclear hole, indicating that the labeled neuron was in the plane of section. Areas of interest were drawn manually around the area containing the VTA+SN (outlines), and all of the nGFP and TH-immunoreactive cells in the left hemisphere of these structures were counted. ***A***, RV nGFP and TH labeling in the rostral VTA/SN. In the region of interest outlined, 9/1606 DAPI-labeled cells were labeled with GFP (0.56%). ***B***, ***C***, RV nGFP and TH labeling in the caudal VTA/SN. In the region of interest outlined, 83/2595 DAPI-labeled cells were labeled with GFP (3.2%). Co-localization of nGFP and TH was very rarely observed. In ***C***, the arrow indicates an example of an nGFP-labeled nucleus which may be in a TH-labeled neuron. The arrowhead indicates an nGFP nucleus which overlies TH-labeling but does not appear to be the nucleus of the TH-expressing cell. ***D***, Count of nGFP labeled, TH-positive, and dual-labeled neurons in the left VTA/SN in sections at the designated coordinates in Case r1. IP, interpeduncular nucleus; ml, medial lemniscus; MM, medial mammillary nucleus; PN, paranigral nucleus; SNC, substantia nigra, pars compacta; SNR, substantia nigra, pars reticulata; VTA, ventral tegmental area. Scale bar: 200 μm (***A***).

To assess for dopaminergic afferents to the IP, we then examined the tegmental dopamine system of the *Chrna5^Cre^* RV-traced mice, including the entire rostrocaudal extent of the SN and VTA, for the expression of RV-nGFP. TH immunostaining was used to identify dopaminergic neurons. Since TH is cytoplasmic, and the RV-expressed GFP is nuclear, we looked for neurons that showed a circular or semicircular pattern of TH staining with a nuclear “hole” indicating that the cell nucleus was in the plane of section. Dopaminergic neurons projecting to the IP should exhibit a “fried egg” appearance with a nGFP-positive nucleus and a TH-immunoreactive periphery. Overall, RV-nGFP labeled neurons were sparse in the VTA (Fig. 2*A*,*B*). They appeared at a somewhat higher frequency in the caudal part of the SN (Fig. 2*B*). Few, if any, of the nGFP-labeled neurons appeared to be dopaminergic. Using Z-stacked confocal images, we counted RV-nGFP labeled neurons and TH-immunoreactive neurons in one hemisphere of the tegmentum from bregma −2.8 to bregma −3.6 (in Cases r1 and r1), encompassing the entire extent of the VTA+SN a standard atlas (Paxinos and Franklin, 2001). RV-nGFP neurons were counted within a manually outlined area of interest encompassing all of the VTA+SN TH-immunoreactive cell bodies (Fig. 2*A*,*B*). TH immunostaining that did not incorporate a nuclear profile was assumed to be contributed by fibers of passage or by neurons with nuclei out of the plane of section and was ignored in cell counts. Figure 2*C* shows an example of a rarely encountered TH-positive neuron that may have a GFP-positive nucleus, and an example of a GFP-positive nucleus that overlaps TH signal in the image but does not appear to be the nucleus of a TH-labeled cell. In Case r1 we counted 1104 TH-immunoreactive neurons in the series, and 373 nGFP labeled nuclei within the area of interest defined by the TH staining (Fig. 2*D*). In 1099/1104 TH-immunoreactive neurons, a GFP-labeled nucleus could be excluded; only 5 cells showed sufficient overlap to suggest possible co-localization of the markers. Results for Case r2 were similar, with only one cell showing possible co-localization (Table 1). We conclude that DA neurons in the SN/VTA very rarely make contact with α5-expressing neurons in the IP. Labeling data for Cases r1 and r2 across the rostrocaudal extent of the VTA/SN appear in Table 1.

**Table 1:** Cell counts for RV-labeling and TH immunoreactivity in Cases r1 and r2

Bregma Coordinate	Case r1 cell count			Case r2 cell count		
	nGFP	TH	Both	nGFP	TH	Both
−2.8	12	43	0	2	28	0
−2.9	9	116	0	2	65	0
−3.0	24	134	1	15	112	0
−3.1	31	117	0	19	102	0
−3.2	52	192	1	39	110	0
−3.3	54	156	1	38	121	0
−3.4	83	158	1	38	145	1
−3.5	59	101	1	36	138	0
−3.6	49	87	0	33	128	0
Sum	373	1104	5	222	949	1

**Figure 3.**
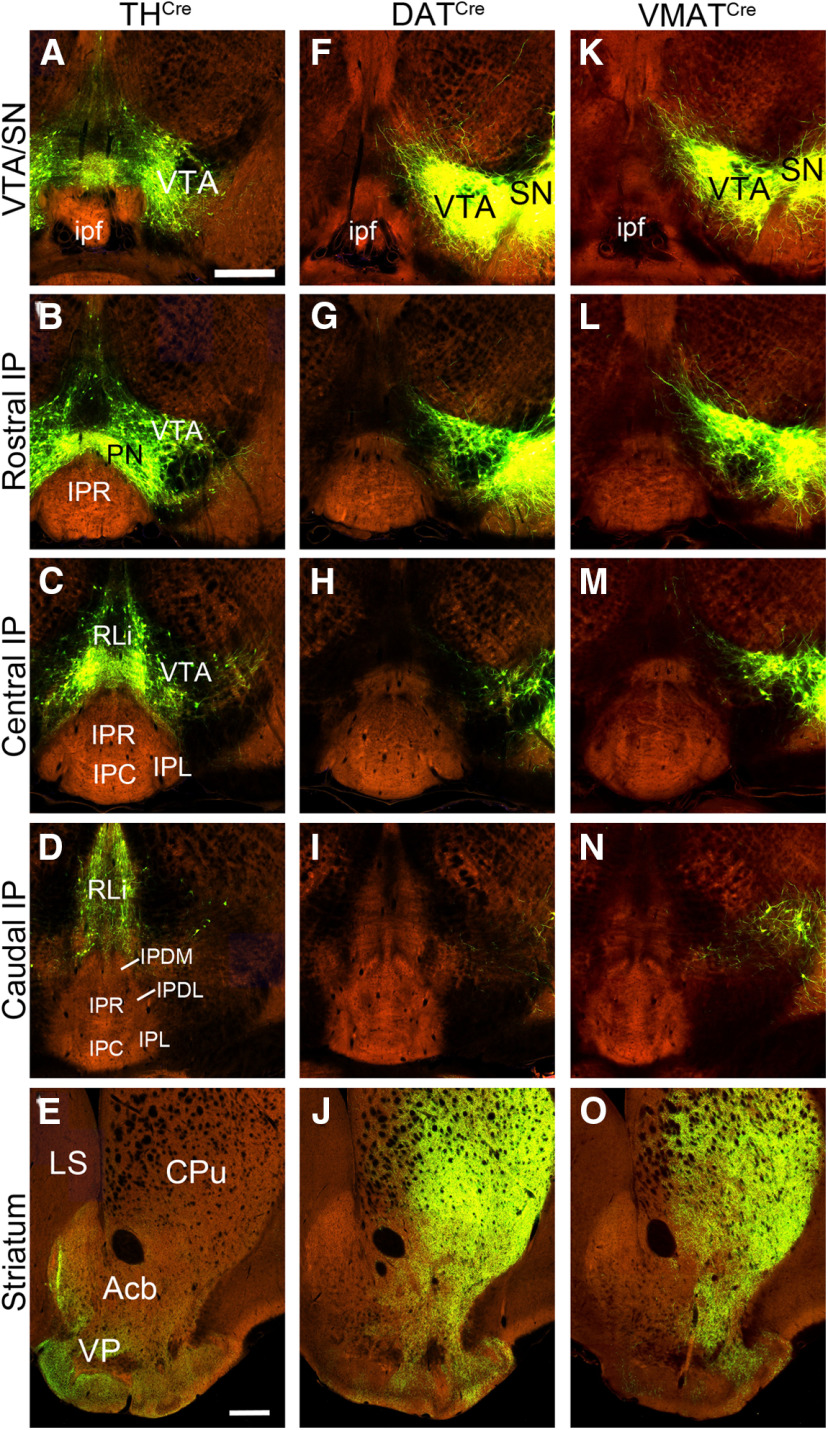
Cre-mediated tract-tracing of tegmental DA neurons in the Allen Connectivity Atlas. Three cases of Cre-mediated AAV tract tracing from the Allen Connectivity Atlas are shown. ***A–E***, Labeling mediated by *TH^Cre^*, Allen experiment 156314762, with labeled neurons in the bilateral VTA, PN, and RLi, and sparing the SN. ***F–J***, Labeling mediated by *Slc6a3^Cre^* (*DAT^Cre^*), Allen experiment 160539283, with signal in the right VTA and SN. ***K–O*,** Labeling using a *VMAT2^Cre^* driver, Allen experiment 292958638, with expression in the right VTA and SN. The planes of section from top to bottom are: (***A***, ***F***, ***K***) VTA/SN, incorporating the injected area, close to bregma −3.1 as designated in a standard atlas (Paxinos and Franklin, 2001); (***B***, ***G***, ***L***) rostral IP, bregma −3.3; (***C***, ***H***, ***M***) central IP, bregma −3.5; (***D***, ***I***, ***N***) caudal IP, bregma −3.8; (***E***, ***J***, ***O***) striatum, bregma 0.7. The reported target coordinates are (bregma, AP, ML, DV): experiment 156314762 (−3.28, 0.36, 4.13); experiment 160539283 (−3.08, 1.25, 4.08); experiment 292958638 (−3.08, 1.25, 4.15). Acb, accumbens nucleus; CPu, caudate/putamen; IP, interpeduncular nucleus; IPC, central part; IPDL, dorsolateral part; IPDM, dorsomedial part; IPL, lateral part; IPR, rostral part; ipf, interpeduncular fossa; LS, lateral septum; PN, paranigral nucleus; RLi, rostral linear nucleus raphe; SN, substantia nigra; VP, ventral pallidum; VTA, ventral tegmental area. Scale bar: 400 μm (***A***, ***E***).

Although most IP neurons express *Chrna5^Cre^*, we also considered the possibility that DA neurons in the VTA could project exclusively to a subset of IP neurons that are α5-negative, and thus would not be labeled in the retrograde tracing experiments. In addition, it is possible that RV does not efficiently label all kinds of presynaptic neurons (Rogers and Beier, 2021). For these reasons, we examined data available in a public database addressing the projections of tegmental DA neurons, and performed further Cre-mediated anterograde tracing experiments. The Allen Connectivity Atlas is a large, searchable database of cases in which the mouse brain has been injected with AAV tracers, then imaged by serial two-photon tomography through the entire neural axis (Oh et al., 2014). Many of the Allen Connectivity Atlas experiments use Cre-dependent AAV combined with mouse strains expressing Cre recombinase in specific populations of neurons. Allen cases mapping the efferents of the SN/VTA are available using three Cre-drivers for catecholaminergic neurons: *TH-IRES^CreER^* (*TH^Cre^*), *Slc6a3^Cre^* (*DAT^Cre^*), and *Slc18a2^Cre^* (*VMAT2^Cre^*). We thus examined AAV-GFP expression in the SN/VTA and IP of each of these transgenic models (Fig. 3). The use of multiple transgenic models helps to overcome possible heterogeneity in the labeling of DA neurons in different transgenic systems (Lammel et al., 2015; Stuber et al., 2015). As a positive control for the successful labeling of efferents from the tegmental DA system, we examined labeled fibers in the striatum in each case. The available TH^Cre^ case, injected near the midline, labeled DA neurons predominantly in the caudal VTA, as well as DA neurons of the paranigral nucleus (PN), and the rostral linear nucleus raphe (RLi; Fig. 3*A–D*). As expected in a case that did not label the SN, fibers in the striatum terminated mainly in the ventral pallidum (VP), rather than the caudate/putamen (CPu; Fig. 3*E*). Two *DAT^Cre^* mediated VTA-labeling cases are available in the Allen database, and the more extensively labeled case is shown (Fig. 3*F–J*). In this case, injected unilaterally in the right tegmentum, there are numerous labeled cell bodies in the rostral and central parts of the SN and VTA (Fig. 3*F–H*). Terminal fibers are seen in both the ventral (VP, Acb) and dorsal (CPu) striatum. A reported case using a *VMAT^Cre^* mouse strain shows labeling of cell bodies in the SN/VTA and terminal fibers in the striatum that is very similar to that obtained with *DAT^Cre^* (Fig. 3*K–O*). Taken together, these three cases gave good coverage of DA neurons in the tegmentum and their striatal targets of innervation. No case showed evidence of dopaminergic innervation of the IP (Fig. 3*B–D*,*G–I*,*L–N*).

**Figure 4.**
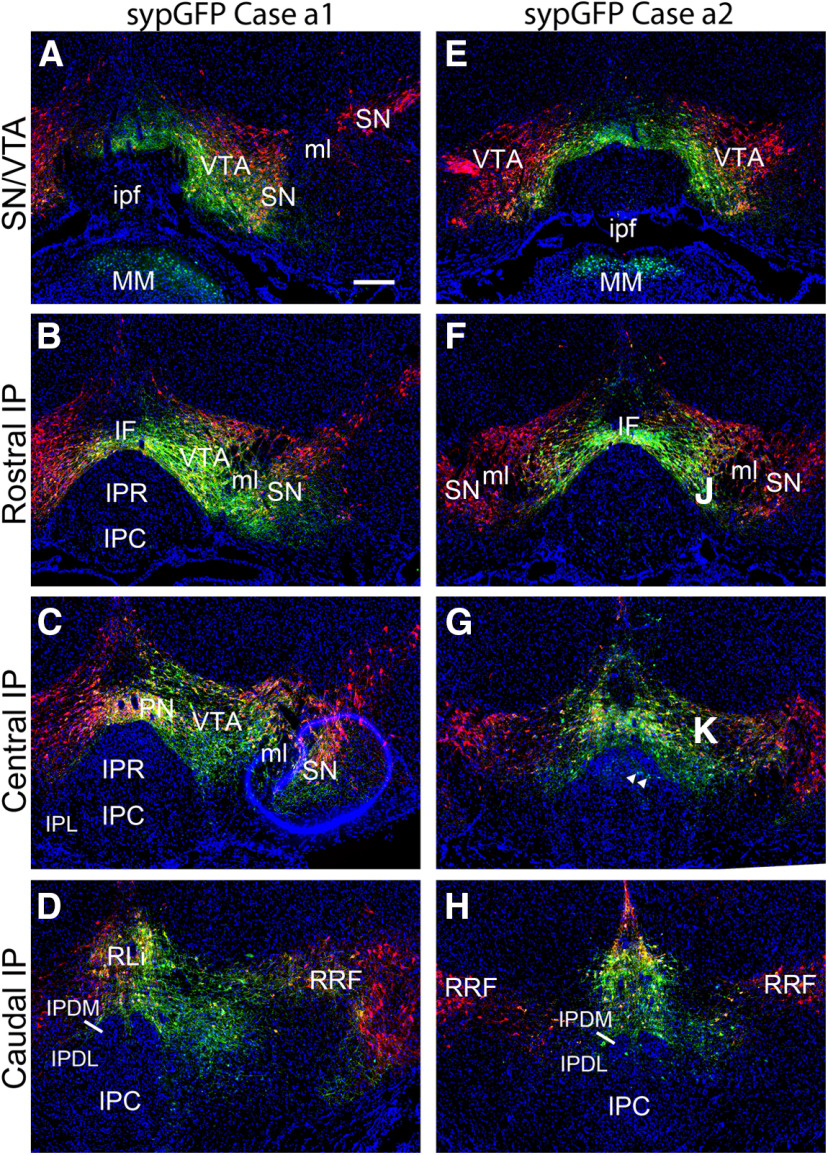
Cre-mediated tract tracing of tegmental DA neurons using synaptically-targeted GFP. *DAT^Cre^* mice were injected with a Cre-dependent AAV expressing synaptically targeted GFP. Sections were stained for TH by immunofluorescence (red), and with the nuclear marker DAPI (blue). The intended target of the injection was at the level shown in ***B***, ***F***. The targeted coordinates for both cases were: AP −3.4, ML 0.5, DV 4.5. ***A–D***, Case a1 (anterograde 1), injected in the central part of the right VTA, with some spread laterally into SN and RRF. ***E–H***, Case a2, injected nearer the midline, labeling the medial part of the VTA bilaterally, as well as the PN and RLi. Arrows in ***G*** indicate rare cell bodies in the IP labeled by AAV, indicating that a few IP cells may express enough Cre recombinase to activate the Cre-dependent AAV. The planes of section from top to bottom are: (***A***, ***E***) injected area, bregma −3.1; (***B***, ***F***) rostral IP, bregma −3.3; (***C***, ***G***) central IP, bregma −3.5; (***D***, ***H***) caudal IP, bregma −3.8. IF, interfascicular nucleus; IP, interpeduncular nucleus; IPC, central part; IPDL, dorsolateral part; IPDM, dorsomedial part; IPL, lateral part; IPR, rostral part; ipf, interpeduncular fossa; ml, medial lemniscus; PN, paranigral nucleus; RLi, rostral linear nucleus raphe; RRF, retrorubral field; SN, substantia nigra; VTA, ventral tegmental area. Scale bar: 200 μm (***A***).

To complement these tract-tracing cases available in a public database, we performed independent injections in *DAT^Cre^* mice targeting the central to caudal VTA, which were relatively under-labeled in the Allen *DAT^Cre^* cases (Fig. 4). In order to enhance detection of any projections from tegmental DA neurons to the IP, we used a Cre-dependent AAV expressing synaptically targeted GFP (sypGFP), which produces intense punctate signal in presynaptic areas. Serial sections through the tegmentum of the injected cases were immunostained for TH to visualize the DA cell bodies. Case a1 was a unilateral injection in the central/caudal VTA, also labeling the PN, and largely sparing the SN (Fig. 4*A–D*). Case a2 was a midline injection labeling both VTA hemispheres, the PN, and the RLi (Fig. 4*E–H*). Intensely labeled DA neuron cell bodies were observed in all of the injected areas. Rare GFP-labeled cell bodies were also detected in the caudal IP, indicating that a few IP cells express *DAT^Cre^* (Fig. 4*G*). These cells were not TH immunoreactive and their identity is unclear. However, no sypGFP labeling was detected in the IP indicative of dopaminergic afferents.

## Discussion

Two recent papers have proposed a novel and behaviorally significant connection between tegmental DA neurons and the IP (Zhao-Shea et al., 2015; Molas et al., 2017). Certainly, new pathways may be discovered linking well-studied brain regions. However, the projections of the midbrain DA system have been extensively studied in rats, mice, primates, and other species, so it is surprising that such a pathway, if present, has not been previously reported. Indeed, the mesolimbic and nigrostriatal DA pathways may be the most-studied subcortical systems. A comprehensive review of the first ∼170 studies of the VTA efferent system in birds, rodents, and primates makes no mention of a VTA-IP projection (Oades and Halliday, 1987), nor do subsequent reviews of studies using classical methods (Bentivoglio and Morelli, 2005). Further studies of this system using a wide range of genetic, viral, and optogenetic methods, and reviews of those studies, also do not mention projections of tegmental DA neurons to the IP (Yetnikoff et al., 2014; Aransay et al., 2015; Beier et al., 2015; Wise and McDevitt, 2018). Likewise, a viral anterograde study of GABAergic and glutamatergic VTA neurons did not identify IP projections (Taylor et al., 2014).

Although the existing IP literature is much sparser, prior studies of global inputs to the IP also have not described a VTA-IP dopaminergic connection. Brain-wide studies of projections to the IP using classical methods in the rat have not identified VTA or SN neurons projecting to the IP (Marchand et al., 1980; Groenewegen et al., 1986; Shibata et al., 1986). A recent study using retrograde CTB tracing from the IP in rats showed rare labeling in the VTA, similar to that observed here using RV, but these neurons were not identified as dopaminergic (Lima et al., 2017). A prior Cre-mediated RV retrograde tracing study of the specific inputs to populations of IP neurons expressing Amigo1 and Epyc, each of which identifies a subset of the α5 nicotinic receptor-expressing neurons labeled in the present study, also did not report VTA labeling from the IP (Ables et al., 2017).

In the present study, we have used two different Cre-driven transgenic strategies in mice to search for connections between tegmental DA neurons and the IP. First, we used a well described BAC-Cre transgenic line, *Chrna5^Cre^* (Morton et al., 2018), to express RV as a specific retrograde tracer in the habenula-recipient subnuclei of the IP. No significant RV labeling from the IP was observed in tegmental DA neurons, although other neurons in the VTA were sparsely labeled. The habenula, in contrast, was heavily labeled by RV via the well-known habenulopeduncular pathway. Second, Cre-mediated anterograde tracing was performed by injection of a Cre-dependent AAV expressing a synaptically-targeted GFP marker into the VTA of a *DAT^Cre^* transgenic line. Despite the enhanced sensitivity obtained by concentrating the GFP label in presynaptic areas, essentially no dopaminergic fibers were identified in the IP.

Given these findings, we also searched for supporting data from other transgenic models in the Allen Connectivity Atlas. Useful experiments were found in the Atlas using three other mouse strains used to identify DA neurons: *TH^Cre^*, *VMAT2^Cre^*, and a different *DAT^Cre^* transgenic line from that used in our own experiments. AAV injections into the tegmentum in these experiments labeled DA neurons in the VTA, as well as other tegmental DA neurons in the SN, PN, and RLi. Although none of these cases, taken alone, completely labeled all of these structures, together they gave extensive coverage of the tegmental DA system. In each case, as expected, dopaminergic projections to the striatum were heavily labeled, but none of these cases showed significant fiber labeling in the IP. These experiments were published online as part of the Allen Connectivity Atlas between October 2012 and March 2014, so they were publicly available at the time the results of Molas et al. (2017) were reviewed and published.

Surprising new findings in a well-studied system should be supported by strong evidence. However, in the published work reporting a VTA-IP pathway (Molas et al., 2017), the evidence for dopaminergic projections to the IP is quite limited. In these experiments, Cre-dependent AAV encoding ChR2-eYFP was injected into the VTA of the same strain of *DAT^Cre^* mice used to generate the data shown in Figure 2. Only a single VTA injected case is shown, in which a few labeled fibers appear in the periphery of the IP (Molas et al., 2017; see their Supplementary Fig. 10). The origin of these fibers is not well defined, because no data were shown to delineate the extent of viral infection. The VTA injections performed in this report employed 800 nl of AAV, a large volume relative to subcortical structures in the mouse CNS. This is in contrast to the 100-nl injection volume used in the present study, and the focused iontophoretic injections used in the Allen Connectivity Atlas experiments. Thus, it is unclear whether the injection was restricted to the VTA, might have spread to other DAT-expressing areas, or could have infected the TH-negative *DAT^Cre^* labeled cells of unknown phenotype that are intrinsic to the IP (Fig. 4*G*). These authors also cite prior work from the same laboratory as support for a VTA-IP projection (Zhao-Shea et al., 2015). In that paper, supported by a single image (see their Supplementary Fig. 9), the authors state that the VTA projects to the centrally located IP subnuclei, IPI and IPC, rather than the periphery of the rostral IP, an entirely different pattern of innervation from that described in the 2017 paper.

In summary, we are unable to reconcile the current findings with the hypothesis that VTA DA neurons have direct input to the IP, and thus mediate behavioral responses to social novelty by this pathway, as proposed. Likewise, there do not appear to be IP inputs from the other next-closest tegmental DA cell groups in the SN, PN, or RLi. Thus, the conclusion that there is a direct functional intersection of the DA reinforcement pathway and the habenulopeduncular pathway at the level of the IP should be reconsidered unless better evidence for such a connection can be found.

## Synthesis

Reviewing Editor: Alexxai Kravitz, Washington University in St. Louis Decisions are customarily a result of the Reviewing Editor and the peer reviewers coming together and discussing their recommendations until a consensus is reached. When revisions are invited, a fact-based synthesis statement explaining their decision and outlining what is needed to prepare a revision will be listed below. The following reviewer(s) agreed to reveal their identity: Martin Metzger.

Essential comments:

1) The reviewers commented that the experimental ns were low (as few as 2 brains in the case of rabies). The reviewers did not think it is essential to increases this number before publication, but the stressed that it would greatly strengthen the paper to analyze a larger sample of brains if possible. On this point Reviewer 3 noted that “the argument that the Tapper papers rely on single micrographs is undercut a bit by the fact that the present paper also appears to rely on a limited number of images from 1 or 2 mice. Can more quantification be presented? The paper states that 5 mice were injected but results from 2 are shown. What do the data look like for the other 3?"

Related, authors should formally summarize and perform statistical analysis on the cell counts provided in Fig 2D, OR with only n=2 cases, they show the individual counts for each case.

2) The efficiency of rabies labeling is variable and factors affecting the tropism are not well understood. This makes it difficult to ‘prove a negative’ by the absence of RV-labeling. Although the authors observed monosynaptic RV-labeled inputs in other known projection regions to IP (demonstrating their rabies strategy worked) it is conceivable that dopaminergic axons (perhaps because they do not form classical synapses) are resistant to labeling via the RV strategy. This limitation should be mentioned in the text. The authors may also want to discuss a recent paper highlighting considerations with RV: https://doi.org/10.1016/j.jneumeth.2020.109005

3) Additional close-up images of the IPN should be included in the figures.

4) The authors state that all IPN subnuclei contain Chrna5(+) cells, but that does not mean that all IPN neurons express Chrna5. Due to this caveat of using the Chrna5-cre line for pan-neuronal labeling of the IPN, the authors should limit their conclusions to state that there does not appear to be evidence for VTA projections to Chrna5(+) IPN neurons. Alternatively, the authors could present additional data from other genetically-defined cell groups that are distinct from Chrna5(+) cells.

5) Additional literature should be considered for inclusion:

When the authors describe the dual glutamatergic/cholinergic nature of ventral MHb neurons that project to the IP they might consider to cite a paper by Frahm et al. 2015, doi:10.7554/eLife.11396

Introduction page 4, second paragraph: The authors might also cite a paper by Sego et al. 2014, doi:10.1002/cne.23533 when talking about LHb projections to the mesopontine raphe.

Introduction p. 4, second paragraph, last phrase: The citation Metzger et al. 2019 should read: Metzger et al. 2021, and Metzger et al. 2019 (a online version) should also be skipped from the References.

Results, p. 11, or Discussion. When discussing the specificity of transsynaptic retrograde labeling, the authors might cite a recent paper by Rogers & Beier, 2021, https://doi.org/10.1016/j.jneumeth.2020.109005

6) Finally, the authors should also consider the full comments made by the reviewers, appended below. While not “essential", these comments should be addressed where possible.

*******************

Reviewer 1

*******************

Even without providing new findings, the paper entitled “Genetically targeted connectivity tracing excludes dopaminergic inputs to the interpeduncular nucleus from the ventral tegmentum and substantia nigra” is highly relevant by carefully trying to reconcile the findings from conventional tracing studies with those from recent viral tracing approaches. In the present paper the authors carefully challenge the findings from two recent studies describing a direct dopaminergic input from the ventral tegmental area (VTA) to the interpeduncular nucleus (IP), a structure now recognized as a major player in behavioral responses to nicotine. When published the aforementioned two papers indeed raised considerable doubts in specialists, since such a pathway has not been described before in a bulk of meticulously conducted tracing studies using conventional tracers. And importantly, this is only one example of unexpected findings obtained with genetic tracing methods (often published in high impact journals), which are hardly to reconcile with the existing literature. Moreover, as the authors state in the abstract, the existence of such a VTA-IP pathway would indeed impact the prevailing model of IP function.

In all the present paper is well written, the experiments are carefully conducted and the findings are discussed with lot of insight. The authors use several appropriate genetic approaches to shed light on the connections of the IP and also use a public data base (the Allen Mouse Brain Connectivity Atlas) to convince the reader that there are, if any, only extremely weak projections from the VTA-nigra complex to the IP, which is fully in line with the existing literature. All the methods used are described in great detail, and the findings are nicely illustrated.

However, I like to raise some minor points and make some suggestions hat should be incorporated in the present manuscript.

Introduction: First phrase, I think it is somewhat more informative to describe the habenula as a “bilateral epithalamic structure” than a “dorsal diencephalic nucleus” as the authors do.

Introduction page 3: When the authors describe the dual glutamatergic/cholinergic nature of ventral MHb neurons that project to the IP they might consider to cite a paper by Frahm et al. 2015, doi:10.7554/eLife.11396

Introduction page 4, second paragraph: The authors might also cite a paper by Sego et al. 2014, doi:10.1002/cne.23533 when talking about LHb projections to the mesopontine raphe.

Introduction p. 4, second paragraph, last phrase: The citation Metzger et al. 2019 should read: Metzger et al. 2021, and Metzger et al. 2019 (a online version) should also be skipped from the References.

Introduction p. 5, second phrase: The beginning of the phrase might better read: Despite these well-established findings, it has been........

Results, middle of p. 9: When describing the partial labeling of the MHbD and LHb, it would be of interest to inform the reader which subregions of the LHb were labeled.

Results, last phrase of p. 10 might better read: Only in 5 cells we observed a partly overlapping pattern of GFP and TH labeling, indicating a possible co-localization of the two markers.

Results, p. 11, or Discussion. When discussing the specificity of transsynaptic retrograde labeling, the authors might cite a recent paper by Rogers & Beier, 2021, https://doi.org/10.1016/j.jneumeth.2020.109005

Discussion: Is something known about a possible dopaminergic input from the retrorubral field (RRF or A8 dopamine cell group) to the IP? If not, this region might be included in the list of DA cell groups not projecting to IP listed in the summary.

In all, this manuscript is a valuable contribution to the growing literature on the IP, highlighting the need to reconcile findings from viral tracing with those obtained with non-genetic tracing methods and underpinning the importance of adequate controls in viral tracing approaches.

*******************

Reviewer 2

*******************

In this work, the authors test the hypothesis that dopamine neurons from the VTA directly innervate the IP. Contrary to this hypothesis, the authors find no evidence for direct dopaminergic projections from VTA to IP, using two convergent approaches: viral retro- and anterograde tracing. These experimental ns are low (2 brains in the case of rabies), which is the main reservation I have with the study. However, the authors are transparent about this and present each case in the figures. Moreover, the authors use existing public databases (allen brain atlas) to bolster their findings. Altogether, I think this study is appropriate for publication in eNeuro, and it is paramount to have accurate anatomical data in the scientific record. The authors discuss recent work that has proposed this pathway to exist and to be important for anti-anxiolytic effects of nicotine. Therefore, this is a potentially important finding and seeks to prevent evidence contrary to prior publications, in order to correct the scientific record, which is firmly within the scope of eNeuro.

My main comment, in addition to the low number of cases is that the efficiency of rabies labeling is variable, and factors affecting the tropism are not well understood. This makes it increasingly difficult to ‘prove a negative’ with an absence of RV-labeling. Although the authors observed monosynaptic RV-labeled inputs in other known projection regions to IP, it is conceivable that dopaminergic axons (perhaps because they do not form classical synapses) are resistant to labeling via the RV strategy. This is a minor concern, as the authors present substantiating pieces of evidence through new experiments and through careful examination of existing brain atlas databases. However, this limitation should be mentioned in the text.

At the end of the discussion (pg. 16), the authors state: “We are unable to reconcile the current findings with the hypothesis that VTA DA neurons have a direct synaptic input to the IP, and thus mediate behavioral responses to social novelty by this pathway, as proposed”. The authors should remove the last clause ‘and thus mediate behavioral responses to social novelty by this pathway, as proposed’. While technically a sound logical extension, the conclusions of the manuscript is stronger if limited to the new data generated.

Authors should formally summarize and perform statistical analysis on the cell counts provided in Fig 2D, OR with only n=2 cases, they show the individual counts for each case.

Minor language suggestion (i.e. Page 5): Consider using “crossing” vs. ‘interbreeding’
